# A large scale study of portable sweat test sensor for accurate, non-invasive and rapid COVID-19 screening based on volatile compound marker detection

**DOI:** 10.1038/s41598-024-68250-9

**Published:** 2024-08-30

**Authors:** Isaya Thaveesangsakulthai, Kaywalee Chatdarong, Naraporn Somboonna, Nuttapon Pombubpa, Tanapat Palaga, Sureerat Makmuang, Kanet Wongravee, Voravee Hoven, Pakpum Somboon, Pattama Torvorapanit, Thumnoon Nhujak, Chadin Kulsing

**Affiliations:** 1 Department of Chemistry, Faculty of Science, Chulalongkorn University, Bangkok, 10330 Thailand; 2 Department of Obstetrics, Gynaecology and Reproduction, Faculty of Veterinary Science, Chulalongkorn University, Bangkok, Thailand; 3 Department of Microbiology, Faculty of Science, Chulalongkorn University, Bangkok, 10330 Thailand; 4 Microbiome Research Unit for Probiotics in Food and Cosmetics, Chulalongkorn University, Bangkok, 10330 Thailand; 5 Sensor Research Unit (SRU), Department of Chemistry, Faculty of Science, Chulalongkorn University, Bangkok 10330, Thailand; 6 Center of Excellence in Materials and Bio-Interfaces, Chulalongkorn University, Phayathai Road, Pathumwan, Bangkok, 10330 Thailand; 7 Department of Electrical Engineering, Faculty of Engineering, Chulalongkorn University, Bangkok, 10330 Thailand; 8 Division of Infectious Diseases, Department of Medicine, Faculty of Medicine, Chulalongkorn University, Bangkok, Thailand; 9 Thai Red Cross Emerging Infectious Diseases Clinical Center, King Chulalongkorn Memorial Hospital, Bangkok, Thailand; 10 Electrochemistry and Optical Spectroscopy Center of Excellence (EOSCE), Department of Chemistry, Faculty of Science, Chulalongkorn University, Bangkok, 10330 Thailand; 11 Multi-Omics for Functional Products in Food, Cosmetics and Animals Research Unit, Chulalongkorn University, Bangkok, Thailand

**Keywords:** COVID-19 screening, Disease diagnosis, Modified PID, Sweat sensor, VOCs, Biochemistry, Microbiology, Biomarkers, Diseases, Pathogenesis, Chemistry

## Abstract

This study established a novel infield sensing approach based on detection of the volatile compound markers in skin secretions. This was based on analysis of volatile compounds in axillary sweat samples collected from RT-PCR-proven Coronavirus disease 2019 (COVID-19) positive and negative populations using gas chromatography-mass spectrometry (GC–MS). The analysis proposed the possible markers of the monoaromatic compounds and ethyl hexyl acrylate. A portable photo ionization detector (PID) incorporated with the selective material towards the marker compounds was then developed with the pressurized injection approach. This provided the accuracy of 100% in the research phase (n = 125). The developed approach was then applied for screening of 2207 COVID-19 related cases covering the periods of the Alpha, Beta, Delta and Omicron variants of SARS-CoV-2 infection in Bangkok, Thailand. This offered the sensitivity, specificity and accuracy ranges of 92–99, 93–98 and 95–97%, respectively.

## Introduction

The recent global impacts of COVID-19 pandemic are critically affecting people lives and causing enormous disruption into many industries. This subsequently leads to development of several approaches for the diagnosis with real-time reverse transcription-polymerase chain reaction (RT-PCR) recognized as the gold standard along with the most widely applied rapid lateral flow tests for SARS-CoV-2 protein detection. Portable biosensor technology has also been applied based on analysis of biological compounds such as antibodies or viral nucleic acids with different detection methods such as colorimetric or electrochemical approach^1^, as well as wearable devices measuring physiological parameters such as the combined feature of resting heart rate, sleep and activity metrics^2^. Alternatively, analysis of volatile organic compounds (VOC) generated during SARS-CoV-2 infection could offer simple and non-invasive diagnosis possibility. With this approach, GC–MS have been widely applied for identification of these compounds with the statistical analysis applied to differentiate positive and negative sample groups and propose volatile markers. Nonanal and methylpent-2-enal^3^, ethanal, octanal, acetone and methanol^4^ could be the markers in the COVID-19 patient breaths. These compounds could be outcome of complex metabolic procedures of the body in response to the infection of the virus and end up in the breath due to the blood-air exchange in the lungs such as SARS-CoV-2/ACE2 receptor binding decreasing endosome pH and affecting synthesis of proteins further converted to specific volatiles^5^, formation of the specific virus-cell interactions, or the oxidative stress in lung^6^. To this end, portable or infield COVID-19 detection system has been reported based on electronic nose detection of specific compound types^7^. Alternatively, the breath analysis can be performed using a range of MS techniques using different ionization or hyphenation approaches^8^.

Compared with breath, analysis of volatile compounds in skin secretion is more attractive in the aspect of safety with the negligible virus level^9^. Volatile compounds from human skin secretions involve several metabolic pathways in different sources such as eccrine, sebaceous or apocrine glands^10^. Furthermore, human body odor can be correlated with the types and contents of skin bacteria^11^ where sweat compounds can undergo oxidation or interaction with microorganisms producing the volatiles^12^. Comprehensive databases for different microbial volatiles have been constructed^13^. Different metabolisms of COVID-19 patients can be expected^5,6^ as well as the affected innate and adaptive immunity which could enhance bacterial adherence, colonization, growth, and invasion into healthy sites^14,15^. This can lead to different microorganism distribution throughout the bodies^16–19^ which can cause microbial dysbiosis, increased opportunistic pathogens or reduce bacterial diversity especially in respiratory and fecal tracts^20^. There could also be bacteria coinfection with SARS-CoV-2 including, for example, *H. influenzae*, *S. aureus*, *K. pneumoniae*, *M. pneumonia*, *S. pneumoniae*, and *P. aeruginosa*^21,22^. Since the upper respiratory tract microbiomes are in a close contact with skin^16^; subsequently bacterial community change on skin could be expected in association with perhaps characteristic volatile compound profiles in skin secretions of COVID-19 patients, as observed for the characteristic odor compounds identified in cells infected with other viruses previously^23^.

For infield detection, biological detections have been reported due to their sensitivity, e.g. COVID-19 sniffer dogs offering > 90% accuracy within a short diagnostic period^24^. Although the limit of detection (LOD) for VOC could be in the ppt level^25^, such approach involves high cost of training, or uncertainty in target compound recognition. Alternatively, electronic nose (e.g. organic semi-conducting sensors) based on detection of aldehydes and ketones have been reported for SARS-CoV-2 infection screening with the sensitivity of 99–100% and the specificity of 99–100%^26^. Long sampling time (e.g. several hours) was required to result in sufficient signal detectable by the equipment, due to the considerably low sensitivity of the technique (e.g. LOD > 0.1 ppm) as well as difficulty of stability and environmental variation^27–29^. However, the sensitivity can be improved using more expensive electronic nose technologies and pre-concentration units. To this end, a SARS-CoV-2 screening platform called “COVID-19 Air Monitor” was reported which can be installed into a closed room or a vehicle and report the result within every 15–30 min^30^.

The goal of the work is to analyze volatile compounds in sweat samples were analyzed using GC–MS. The work then developed the portable sweat-based COVID-19 screening approach using a photo ionization detector (PID) coupled with the patented filter^31^ for sensitive (LOD of < 0.1 ppm) and selective detection of the marker compounds. This was applied for preliminary screening of people who might be infected with SARS-CoV-2 in Bangkok, Thailand.

## Experimental

### Material and methods

The sample collection from patients was approved by the Central Research Ethics Committee (COA-CREC103/2020) and the Institutional Review Boards at Faculty of Medicine, Chulalongkorn University (IRB no. 897/63), Lerdsin Hospital (IRB no. LH631064), Prasat Neurological Institute (IRB no. 64013) and Central Chest Institute of Thailand (COA No. 020/2564) located in Bangkok and Nonthaburi province, Thailand. We confirm that all methods were performed in accordance with the relevant guidelines and regulations.

### Patient population

The investigate population were aged > 18 years, involved with SARS-CoV-2 infection and categorized with mild or moderate COVID-19, but not severe COVID-19^32^. They did not receive anti-viral medicine within 36 h prior to sample collection. The enrolled consent and clinical information were obtained based on the individual electronic devices, or interviews. The positive samples were collected from COVID-19 patients among four hospitals in Bangkok (King Chulalongkorn Memorial Hospital, Lerdsin Hospital, Hospital of Prasat Neurological Institute and Hospital of Central Chest Institute of Thailand). The negative samples were collected from volunteers without COVID-19 risk within the same areas. The population with underlying of skin disease or exocrine gland dysfunction were excluded in this study.

### Sample collection

Cotton rods (SteriPack, USA, cut into 0.8 × 4 cm each) were treated with an autoclave procedure and kept with sterilized packaging before uses. Each volunteer was requested to place two cotton rods under left axilla and two cotton rods under right axilla and hold them for 15 min. Each set of the two cottons (one from the left and one from the right) containing sweat was inserted into a 20-mL screw thread headspace clear glass vial, closed with a magnetic screw aluminum cap sealed with PTFE/silicone septa (National Scientific, Rockwood, TN, USA). This process was then repeated totally resulting in eight cotton rods collected from each volunteer. To this end, the training set of 64 RT-PCR confirmed positive and 61 RT-PCR confirmed negative samples was obtained during the research phase (December 2020–February 2021). These vials were transported under triple-package with biohazard labelling and cold chain system by trained staffs to laboratory room for code registration. Virus inactivation was conducted by storing the vials containing the cotton rods at 25 °C in biosafety laboratory level 2 (BSL2) at King Chulalongkorn Memorial Hospital, Chulalongkorn University for 48 h. The samples were then kept in the 4 °C refrigerator. Prior to analysis, the vials were kept at room temperature (30 °C) for at least 15 min. For SPME GC–MS analysis, 0.1 µL hexane and nonane (1:1, v/v) in a PCR plastic tube (internal standards) was put into the glass vials containing cottons soaked with sweat.

Within the studied period above, COVID-19 outbreak in Bangkok was mainly occupied with Alpha and Beta variants of SARS-CoV-2 according to the national epidemiological data. We decided to extend and amend protocol of the study to ethical committee of Faculty of Medicine, Chulalongkorn University to collect additional samples for performance testing of volatile compound markers using the developed sensor. Sweat soaked cotton rods from RT-PCR confirmed SARS-CoV-2 infection or positive of rapid antigen test for SARS-CoV-2 patients, who were admitted at hospital and hospitel undertaken by King Chulalongkorn Memorial Hospital, were timely collected as representative samples of Alpha and Beta (March–April 2021), Delta (April–October 2021) and Omicron (November 2021) variants of SARS-CoV-2, respectively. While negative samples were collected from volunteers without any respiratory tract symptoms and the results of rapid antigen tests for SARS-CoV-2 or RT-PCR were negative during each variant period. In summary, 353, 1539 and 315 samples, respectively, with the available consent forms were further obtained to validate performance of our developed sensor in the latter analysis. They are all the test samples. In addition, 130 sweat samples (72 positive and 58 negative samples) covering all the variants from the sample set above were selected for testing with PID-Filter A and PID-Filter B. These samples were then prepared with the same process mentioned above albeit without the internal standard addition.

### Volatile organic compound analysis

Standards of aromatic compounds and n-alkanes (C8–C20) were purchased from Sigma Aldrich (St. Louis, MO). The alkane mixture was used as a reference to calculate the retention indices (I) of the analyte peaks. Styrene, xylene isomers and toluene were obtained from Merck, Germany. A 20 mL glass vial and screw cap vial (Agilent, USA) were used in sample collection process. Solid phase micro extraction (SPME) fiber (50/30 µm DVB/CAR/PDMS, grey) and the holder were purchased from Supelco (Sigma-Aldrich, Bellefonte, PA, USA). The SPME fiber was conditioned at 270 °C for 1 h via insertion into the GC injection port. Prior to sample analysis, the blank fiber was injected to check the background signal from the fiber. The collected sweat samples (2 rods of armpit swab) after the virus inactivation procedure were left in 20 mL glass vials, closed with aluminium caps with sealed PTFE/silicone screw septa. The glass vial contained sample was then left for 5 min equilibrium condition at 70 °C. The SPME fiber was then exposed inside each vial to extract volatile compounds in the headspace sample at 70 °C with 50 min extraction time. The extracted samples were then injected into the GC injection port at 220 °C with the desorption time of 5 min followed by the analysis with GC-QqQMS (7890A-7000, Agilent technologies Inc.). Blank cotton materials were also analyzed under the same experimental condition in order to remove the background signals contributed to the sampling materials.

Volatile organic compounds (VOCs) were separated on a HP-5 MS capillary column (30 m × 0.25 mm inner diameter, 0.25 µm film thickness; J&W Scientific, USA) using ultra-high purity helium (99.999%) as the carrier gas with a flow rate of 1 mL min^−1^. The extracted sample was injected at 220 °C (desorption temperature) under splitless mode. The GC oven temperature was programmed to increase from 40 to 240 °C at a rate of 4 °C min^−1^. MS detector was applied under single Q scan mode with the other quadrupoles operated in total transfer of ion mode. The temperature of the ion source in the MS was set at 230 °C. The electron ionization voltage was − 70 eV. The mass spectra were acquired over the mass range of 30–300 Da with a scan time of 100 ms.

### Analysis with the portable sweat test device

The portable photo ionization detector (Honeywell UltraRAE 3000 + , USA) was applied in this study with the ionization energy of 9.8 eV. Prior to the sweat test, the PID instrument was coupled with a glass tube containing Filter A (RAE-Sep benzene separation tubes, Honeywell) or B which were modified from Filter A^31^. Headspace (10 mL) of a sweat sample collected using the method above was taken into a 10 mL disposable syringe (Terumo, Japan). The sample was then manually pressurized through the filter and further detected with the PID. The instrument was calibrated by using 1 ppm benzene calibrate gas tank (Honeywell) within every 100 injections. All the sample signals were converted into the unit of ppm of benzene. The negative and positive samples were that expected with the signals of 0.00 and > 0.00 ppm benzene. Positive (RT-PCR detected of SARS-CoV-2 or standard benzene derivative samples) and negative controls (RT-PCR not detected of SARS-CoV-2 samples) were also injected within every 10 sample analyses. The same analysis approach using the PID without any filter was also performed for the recovery tests.

### Characterization of the filter materials with portable Raman

Filter material inside a glass tube was directly placed in close contact with the detection chamber (dimension of 155 × 290 × 73 mm) of Resolve handheld Raman analyzer (Agilent, USA) and analyzed using through-barrier scan mode. The instrument was provided by the environment was covered with the radiation protected sheet (Agilent, USA) prior to the measurement with the scan range of 200–2400 cm^−1^. The instrument was calibrated and measured under scan through the mode calibration and performance check.

### Data analysis

Data presentation and peak integration were performed by using Agilent MassHunter software. Chromatographic peaks of interest were tentatively identified by the comparison of their MS spectra with those obtained from the NIST library 2017. The identification criteria were selected with a match score of > 650 and a difference of within 20 units between the calculated retention index (*I*) and the literature I for the same (or a similar) stationary phase. All the results were further processed by using Microsoft Excel. Note that the experimental *I* of each peak was calculated by comparison of the peak retention time with that of an alkane mixture under the same experimental conditions according to the Van den Dool and Kratz relationship. Accuracy rate at a certain threshold of total benzene derive content was determined by the proportion of true positive and true negative in all evaluated samples. This was performed by using Microsoft Excel for all the threshold values of GC–MS peak areas and PID responses in order to construct a receiver operating characteristic (ROC) curve.

## Results and discussion

**Figure 1:**
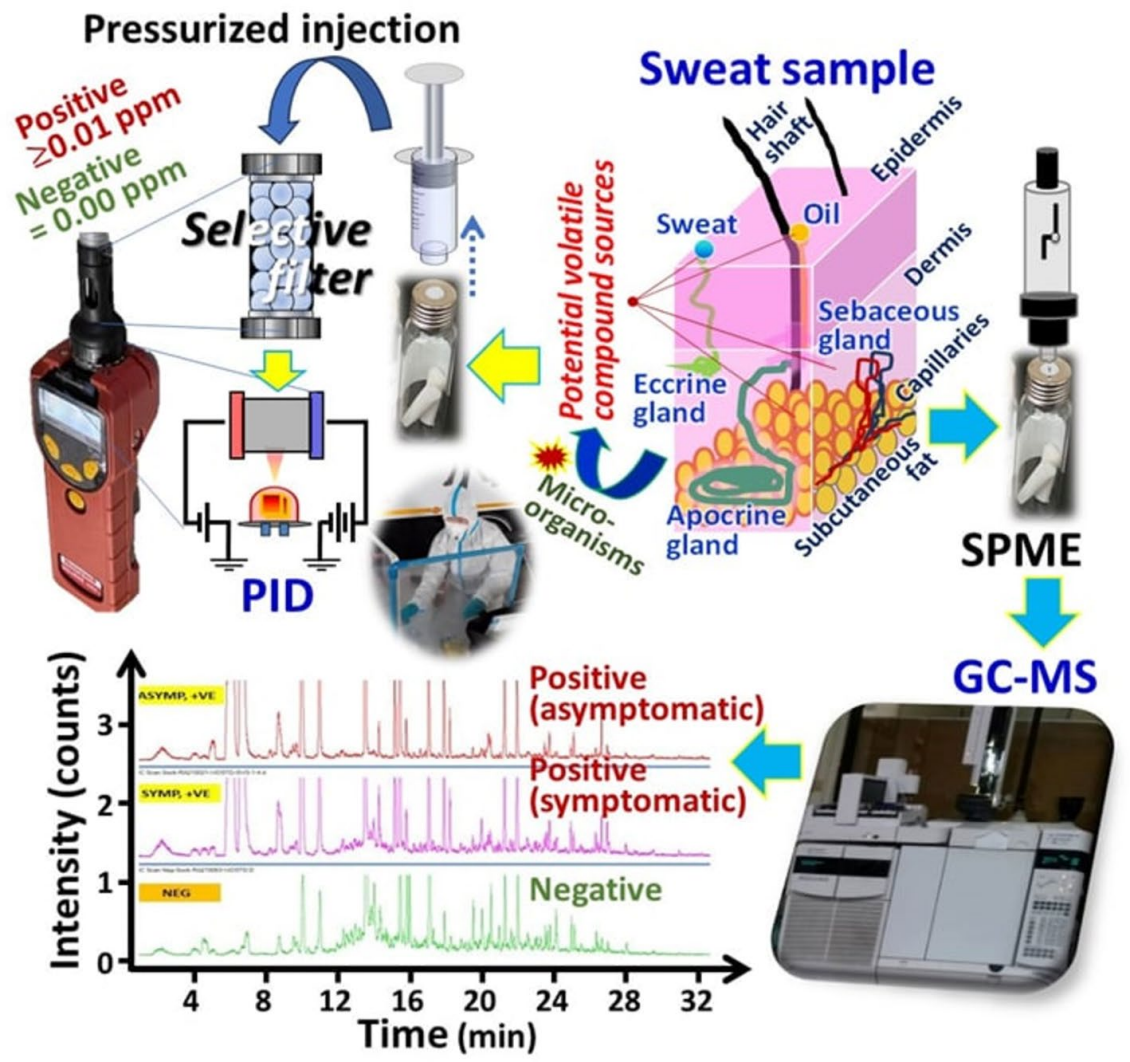
Diagram showing the sweat based COVID-19 screening approaches using SPME GC–MS, with the total ion chromatograms of two COVID-19 positive samples and the negative control, and the direct analysis with PID-selective filter.

Due to the disrupted metabolisms of COVID-19 patients, their sweat volatile compounds are hypothesized to be different compared with the healthy population. This study thus developed volatile compound analysis approaches for COVID-19 screening using SPME GC–MS and direct pressurized injection onto PID coupled with the selective filter as illustrated in Fig. 1. This figure also illustrated the sweat chromatograms belonging to the PCR proven COVID-19 positive patients (both symptomatic and asymptomatic) which are different from that of the negative control confirming the hypothesis above.

Whilst the early study reported with the investigated samples obtained from July 2020 to March 2021 relied on use of electronic nose to perform the screening based on the key markers of aldehydes and ketones^26^, our later investigation with GC–MS analysis of the cotton samples containing armpit sweat during the Alpha and Beta variants revealed additional markers of acetophenone, (E)-2-octenal, 1-chloro-octane and 2-ethylhexyl acrylate investigated with the COVID-19 positive patients in King Chulalongkorn Memorial Hospital, Bangkok, Thailand, unpublished data. Further investigation towards a larger set of armpit sweat samples led to discovery of the potential COVID-19 markers of nonanal (88% accuracy analyzed with a fluorescence-based sensor, n = 85, covering the Alpha, Beta, Delta and Omicron variants)^28^, *p*-cymene (96% accuracy using GC-flame ionization detector, n = 368 covering the Alpha, Beta and Delta variants)^33^, linalool (98% accuracy) and 2,6,11-trimethyldodecane (94% accuracy) both with SPME prior to analysis with comprehensive two dimensional GC (n = 66 covering the Alpha, Beta and Delta variants)^34^, and the combined feature of styrene, xylene, ethylbenzene, nonanal, and 2-ethylhexyl acrylate (94% accuracy with SPME GC–MS, n = 140)^29^. In addition, the study in 2023 reported the Delta and Omicron variants sharing the markers of diacetone alcohol, styrene, 2-pentylfuran, phenylacetaldehyde, undecane, methyl caprylate, trans-2-nonenal, 1-nonanol, decanal, 2-phenoxyethanol, dodecanal, 6,10-dimethyl-5,9-undecadiene-2-one-(e), 1-dodecanol and dodecanoic acid^35^.

Among these compounds, the benzene derivatives could be sensitively detected with a PID with the incorporation of a suitable filter for enhanced selectivity in the sweat sample matrices. The SPME GC–MS data of 125 samples obtained from Chulalongkorn hospital (the training data during the research phase) were analyzed focusing on benzene derivatives with the selected examples with their accuracy data and *p*-values (< 0.05) from the *t*-test to differentiate the COVID-19 positive and negative samples provided in Table 1. These compounds were also targeted by the PID analysis as well as the other compounds with lower accuracy such as ethylbenzene and xylene. The identified benzene derivatives could be produced by several potential bacteria reviewed and some were identified in our study, in Table S1. Further results and discussion related to relationship between benzene derivatives and skin microbiomes and the volatile markers for COVID-19 screening investigated with the GC–MS analysis are provided in supporting information.

**Table 1:** Selected compounds with the statistical parameters to differentiate the positive and negative samples during the research phase (n = 125).

Compound	Accuracy (%)	*p*-value	Average area (× 10^3^ counts⋅s)	
			Positive	Negative
2-Ethyl hexyl acrylate	87	5.39E − 19	12.7 ± 6.1	0.8 ± 1.5
6-methyl 5-hepten-2-one	71	5.99E − 12	4.1 ± 2.1	0.6 ± 1.0
Toluene	73	6.77E − 05	8.8 ± 4	3.8 ± 3.5
Styrene	69	1.08E − 01	55.7 ± 50.1	31.3 ± 43.7
Hexadecanol	66	0.034	4.3 ± 3.7	1.9 ± 3.1
Tridecanal	71	1.69E − 16	3.0 ± 1.7	0.1 ± 0.3
*p*-Cymene	92	1.18E − 16	11.1 ± 5.2	1.2 ± 2.0
Acetophenone	88	1.86E − 25	7.6 ± 3.2	0 ± 0

### PID incorporated with a modified benzene selective filter as a portable COVID-19 sensor

**Figure 2:**
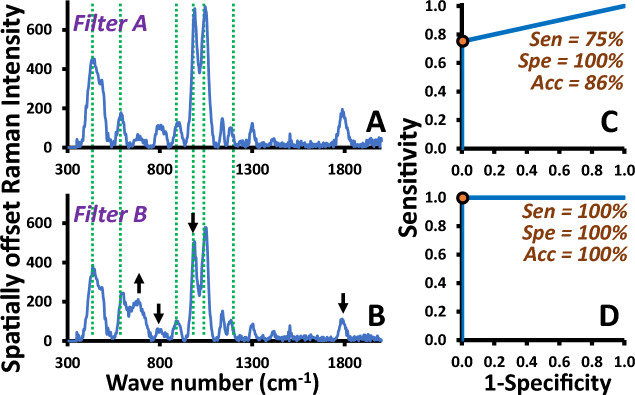
Average portable Raman results of (**A**) Filter A and (**B**) Filter B (n = 3) obtained using Resolve, with the ROC curves obtained from the responses of PID-Filter A and PID-Filter B provided in C and D, respectively. The dots show the optimal cutoff thresholds of the portable sensor responses to discriminate the COVID-19 patients during the period of the delta variant (n = 130).

The overall analysis process with the PID is illustrated in Fig. 1. With this device, a SPME vial containing cottons containing sweat was initially equilibrated at room temperature. This is followed by taking the sample headspace (10 mL) and the subsequent manual injection (thumbed by the researcher and pulled by the pump of the PID instrument) onto the filter. The eluting pulse was then transferred onto a PID showing the signal converted into ppm relative to benzene. The 9.8 eV PID is considerably nonselective which requires separation techniques or selective materials to selectively detect the target volatile markers. A commercial benzene selective filter (Filter A) has been modified using the treatment trademark into Filter B^31^ with selectivity towards different set of the compounds for improved COVID-19 screening performance. Filter A was analyzed by a portable Raman instrument. The characteristic wave numbers for these materials were 443, 591, 903, 988, 1050 and 1200 cm^−1^ (Fig. 2) which indicate the presence of sulfuric acid in aqueous (enabling catalytic oxidation of non-aromatic polar compounds in the sample headspace) as well as the supporting material (carbon based porous polymer Tenax@). The modification process led to the peak intensity being reduced at 801, 988 and 1798 cm^−1^ and increased at 682 cm^−1^ in Filters B. This may be related to conversion of sulfuric acid and the material as well as addition of chemical species in the treatment process. Note that the purpose behind the modification was to selectively alter the catalytic oxidation/reaction selectivity as well as adjusting the polarity of the material towards releasing more marker compounds into the PID after passing the sample through Filter B.

**Figure 3:**
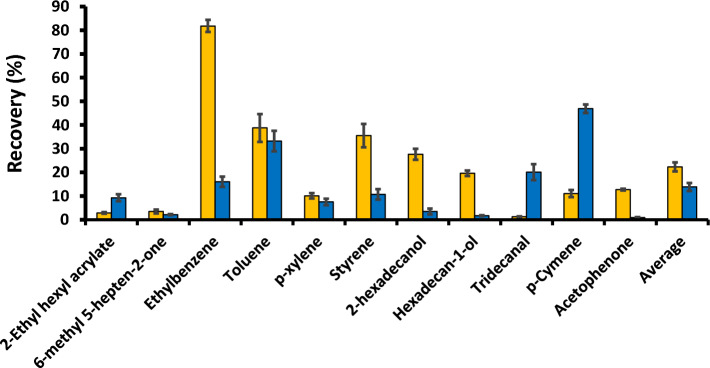
Recoveries of different compounds measured by the PID coupled with *Filter A* (yellow) and *Filter B* (blue): left and right, respectively, relative to the signal measurement without the filter. The relatively standard deviation data of *Filter A* and *Filter B* were within the range of 3–22% and 4–34%, respectively.

For the performance tests, the plots of recoveries of different compounds in sweat from these materials are provided in Fig. 3. The unmodified material (Filter A) expectedly provides high selectivity towards only benzene under atmospheric sampling condition, e.g. with the other derivatives showing < 1% recoveries from the filter^36^. However, under pressurized injection (10 mL) applied in this study, different selectivity was observed with the enhanced recoveries towards ethylbenzene, toluene, styrene and the long chain alcohols. Although applications of Filter B reduced the recoveries in average, this filter enabled several marker compounds (2-ethyl hexyl acrylate, tridecanal and *p*-cymene) to pass through the filter and reach the PID with the higher recoveries (Fig. 3).

### Performance of COVID-19 screening approach using the developed sensor

The portable PID coupled with Filter A or B was initially applied to perform COVID-19 screening tests for the set of 130 sweat samples collected during the period of the Alpha, Beta and Delta variants (72 positive and 58 negative samples). These samples were the subset of that described in Sect. "Sample collection". The collected positive and negative samples were validated according to “detected by RT-PCR or rapid antigen test” and “not detected by RT-PCR or rapid antigen test within 14 days after the sweat test”, respectively. With the criteria that the positive samples showing the signals of ≥ 0.01 ppm, Filters A and B showed accuracies of 86% and 100%, respectively, see also the corresponding ROC curves in Fig. 1C and D, respectively. The greater performance of Filter B corresponds to the relatively high recoveries of *p*-cymene and 2-ethylhexyl acrylate (Fig. 3) which are the potential markers supported by the high areas under the ROC curves of these compound in Fig. S1. Filter B was then applied to perform further screening tests. It should be noted that this material is not exclusively selective for all the marker compounds. However, this is sufficient to extract the target compounds within the matrix of the heated headspace of the sweat sample. A challenge is thus to develop material tailored made with the greater recoveries of the expected marker compounds in Fig. 3 in order to improve the screening performance in the future.

Portable PID-Filter B was applied for the screening test of the same sample set during the period of the alpha–beta variants as that investigated with GC–MS above. This resulted in sensitivity of 100% and specificity of 100% (Fig. S2). This performance was greater than that offered from the GC–MS analysis of the individual compound in Fig. S1A–E (≤ 92% accuracy). This can be explained in the way that more marker compounds could pass through the applied filter as confirmed by the result in Fig. S1F where combination of the marker compounds showed the improved screening performance^29^. It is also possible that the sweat matrix reacted with the chemicals in Filter B producing other marker products detectable by the PID.

### Application of the developed sensor for screening of COVID-19 positive populations in Bangkok, Thailand

**Figure 4:**
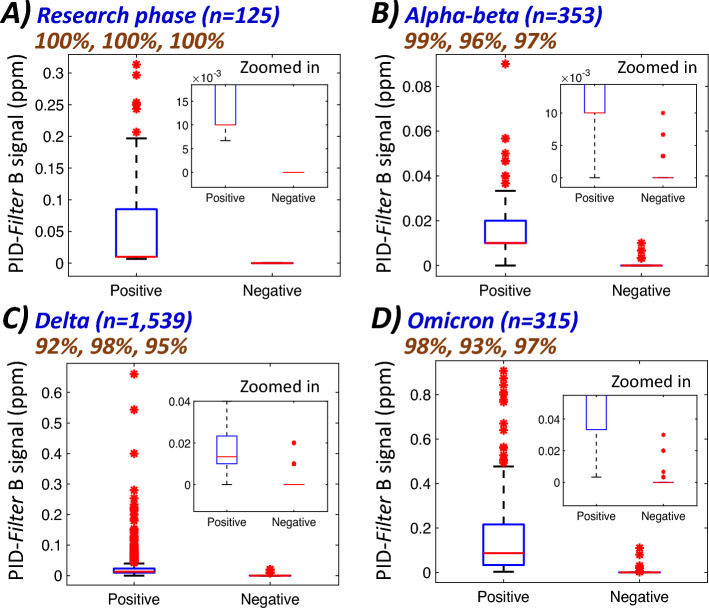
Distribution plots of the developed PID-Filter B signals for the COVID-19 screening tests during the periods of different variants (Research phase, Alpha–beta, Delta and Omicron, **A**–**D**, respectively). The %values from left to right in each figure indicate sensitivity, specificity and accuracy, respectively.

In this section, the collected COVID-19 positive samples; RT-PCR detected or rapid antigen test positive and COVID-19 negative samples; RT-PCR not detected or asymptomatic and rapid antigen test negative, were validated. The related numbers were 64 positive & 61 negative, 156 positive & 197 negative, 684 positive & 855 negative and 253 positive & 62 negative samples during the periods of research phase, Alpha–Beta, Delta and Omicrons variant of SARS-CoV-2 outbreak, respectively. They were investigated with the portable PID-*Filter B* with the signal distributions plotted in Fig. 4 and the ROC curves showing the performances provided in Fig. S2. This indicates ≥ 95% accuracies of the developed approach for screening of the investigated COVID-19 samples in Bangkok. Thus far, more than 8000 cases have been screened.

Acceptable repeated screening capability was also achieved as illustrated by the same positive samples showing the average signal of ≥ 0.01 ppm (Fig. S3) as well as the negative controls showing 0.00 ppm over > 240 screening tests. Note that a positive control can be a set of PCR positive samples; whilst the negative controls were the PCR negative samples. Each sample can be reused for several days without significant signal drops albeit with the requirement of ≥ 5 min of equilibrium time prior to each repeated sampling. Discussion related to false positive and false negative is provided in Supporting information. Limitation of our screening approach is that microbial distribution could be geographically different affecting distribution of the skin volatile compounds. This approach is thus not expected to be effectively applicable everywhere in the world. It should also be noted for an outdoor screening test that some environment such as air pollution, an area with strong smell of food or a new construction with the new painting should be avoided. Moreover, we could not exactly identify the variant of SARS-CoV-2 of all samples due to limited resource and budget. The application of the results among all variants had to be proposed just by national epidemiological data. However, volatilome represented from metabolism and biological reaction of SARS-CoV-2 infection would generally not be affected by spike mutation in theory.

## Conclusions

Novel approach for COVID-19 screening tests has been established based on detection of monoaromatic and the related compound markers in sweat samples collected onto cotton rods. Sample collection process could be performed within 20 min. This is followed by pressurized injection of the sample headspaces into the PID instrument coupled with the selective filter material showing the result within a few seconds. The approach was successfully demonstrated for the screening of 2207 samples with ≥ 95% accuracies. Each analysis cost could be lower than half of a SARS-CoV-2 antigen test for the long-term use (potentially saving ~ 1 MUSD of the cost structure for a million tests). The approach is also non-invasive avoiding the nasal pain as well as generating lower amounts of biohazard wastes.

### Supplementary Information

Supplementary Information.

## Data Availability

The datasets used and/or analyzed during the current available from the corresponding author on reasonable request.
