# Supplementary material for: A large scale study of portable sweat test sensor for accurate, non-invasive and rapid COVID-19 screening based on volatile compound marker detection
Source: Sci Rep. 2024 Aug 30;14:20148. doi: 10.1038/s41598-024-68250-9 (PMC11362290; doi:10.1038/s41598-024-68250-9)
Supplement: Supplementary file 1 — Supplementary Information. [file 41598_2024_68250_MOESM1_ESM.docx]

**Supporting Information (SI)**

**A Large Scale Study of Portable Sweat Test Sensor for Accurate, Non-invasive and Rapid COVID-19 Screening Based on Volatile Compound Marker Detection**

Isaya Thaveesangsakulthai^a^, Kaywalee Chatdarong^b^, Naraporn Somboonna^c,d^, Nuttapon Pombubpa^c,d^, Tanapat Palaga^c^, Sureerat Makmuang^e^, Kanet Wongravee^e^, Voravee Hoven^a,f^, Pakpum Somboon^g^, Pattama Torvorapanit^h,i^, Thumnoon Nhujak^a^ and Chadin Kulsing*^a,j^

*^a^Department of Chemistry, Faculty of Science, Chulalongkorn University, Bangkok 10330, Thailand*

*^b^Department of Obstetrics, Gynaecology and Reproduction, Faculty of Veterinary Science, Chulalongkorn University, Bangkok, Thailand*

*^c^Department of Microbiology, Faculty of Science, Chulalongkorn University, Bangkok 10330, Thailand*

*^d^Microbiome Research Unit for Probiotics in Food and Cosmetics, Chulalongkorn University, Bangkok 10330, Thailand*

*^e^Sensor Research Unit (SRU), Department of Chemistry, Faculty of Science, Chulalongkorn University, Thailand*

*^f^Center of Excellence in Materials and Bio-interfaces, Chulalongkorn University, Phayathai Road, Pathumwan, Bangkok 10330, Thailand*

*^g^Department of Electrical Engineering, Faculty of Engineering, Chulalongkorn University, Bangkok 10330, Thailand*

*^h^Division of Infectious Diseases, Department of Medicine, Faculty of Medicine, Chulalongkorn University, Bangkok, Thailand*

*^i^Thai Red Cross Emerging Infectious Diseases Clinical Center, King Chulalongkorn Memorial Hospital, Bangkok, Thailand*

*^j^Electrochemistry and Optical Spectroscopy Center of Excellence (EOSCE), Department of Chemistry, Faculty of Science, Chulalongkorn University, Bangkok 10330, Thailand.*

* Corresponding author: Dr. Chadin Kulsing

E-mail: Chadin.k@chula.ac.th Tel.: +66 2 218 7721

Results and Discussion

*Relationship between benzene derivatives and skin microbiomes*

This study analyzed cottons containing sweat samples obtained from the positive (RT-PCR or rapid antigen test proven SARS-CoV-2 infection and categorized with mild or moderate COVID-19, but not severe COVID-19 [1] and negative (RT-PCR or rapid antigen test proven) population. The cotton samples were inactivated at 25 °C in a biosafety laboratory level 2 (BSL2) for 48 hours [2]. The samples were analyzed with GC-MS and PID coupled with the selective filter [3]. Since PID could also detect other benzene derivatives, the obtained GC-MS data from the previous work, *in preparation*, were revised focusing on these compounds. The identified benzene derivatives could be produced by several potential bacteria reviewed and some were identified in our study, in **Table S1**. Several of them could be related to COVID-19. For example, *S. epidermidis* on skins could produce xylenes and ethylbenzene [4]. These bacteria were also previously reported in respiratory tract patient with COVID-19 [5]. Toluene can be identified from the skin bacteria such as *B. epidermidis* [6]. Styrene can be used as a marker for *P. aeruginosa* [7,8] which can also be found on skins [9] as well as coinfection of Covid-19 patients [10]. It should be noted that these marker compounds were also reported as the important compounds for the other diseases. Styrene has been reported as a volatile marker for influenza A detection in pig breath along with ethanal, propanal, *n*-propyl acetate, methyl methacrylate and 1,1- dipropoxypropane. [11] Meat spoilage related bacteria such as *Serratia proteamaculans*, *Pseudomonas fragi* and *Carnobacterium maltaromaticum* could also produce xylenes and toluene [9]. It should be noted that benzene derivatives are not likely to be endogenous. For example, they could be produced from bacterial metabolisms [12]. [(*S*)-1-Phenylethanol](http://eawag-bbd.ethz.ch/servlets/pageservlet?ptype=c&compID=c0266) could be metabolized with 1-phenylethanol dehydrogenase to produce acetophenone [13]. Aromatic amino acids (*e.g.* phenylalanine) in sweat [14] could be converted into cinnamic acid by the enzyme phenylalanine ammonia lyase [12]. Cinnamic acid can then be converted into styrene, *e. g.* via the enzyme ferulate decarboxylase and phenylacrylate decarboxylase [12]. Hence, we are in a process of comparing quantitative microbiome profiles between COVID-19 vs. healthy individuals from this study, to support the established reported bacterial species that might underly the characteristic volatile compounds identified by our portable COVID-19 sweat test detector and/or the characteristic odor profiles representing COVID-19 patients.

**Table S1**. Aromatic compounds identified in HS-SPME GC-MS analysis of the samples within the period of Alpha and Beta variants of SARS-CoV-2, the identified bacteria and the references reporting the relationship to SARS-CoV-2 infection. Note that 🗸 indicates the bacteria identified in this study from 10 randomly selected COVID-19 patients.

| **Compound** | **Possible microbiome species** | **Reference** | | |
| --- | --- | --- | --- | --- |
|  |  | **Microbial volatile compound detection** | **Bacteria in human skin** | **Relationship with COVID-19** |
| Toluene | *B. epidermidis* | Peptone C, Yeast extract, Glucose, NaCl and water [6] | [6] | - |
|  | *E. muricatum* | [15]  Only in Philippines (seaweed/food source) and Singapore  [16] | - | - |
|  | *H. erinaceus* | [17] | - | - |
| *o*-xylene | *S. epidermidis* | Brain heart  infusion (BHI) and mueller Hinton broth (MHB) [18] | [6] | [3] |
| *m*-xylene | *E. muricatum* | [15]  Only in Philippines (seaweed/food source) and Singapore  [18] | - | - |
| *p*-xylene | *S. epidermidis* | Brain heart  infusion (BHI) and mueller Hinton broth (MHB) [18] | [6] | [16] |
| Ethyl benzene | *S. epidermidis* | Brain heart  infusion (BHI) and mueller Hinton broth (MHB) [18] | [6] | [16] |
| Styrene | *E. scoparia* | [19] | - | - |
|  | *L. distichophylla* | [19] | - | - |
|  | *P. aeruginosa* | [19] | [6] | [10] |
|  | *P. citreonigrum* | [19] | - | - |
|  | [*S. griseovariabilis*](https://www.amibase.org/detail_data.php?scientific_name=Streptomyces_griseovariabilis) | [15]  Only found in Thailand [19] | - | - |
| 2-phenylethanol | *S. aureus* | 🗸  [22]  [17] | 🗸 | 🗸 [20,21] |
| 1,2,4-trimethyl-benzene | *E. muricatum* | [15]  Only in Philippines and Singapore  [19] | - | - |
| 1,3-dichloro-benzene | *E.muricatum* | [15]  Only in Philippines and Singapore  [19] | - | - |
| Benzaldehyde | *H. Influenzae*  *S. pneumonia* | 🗸  [22] | 🗸  - | 🗸 [20,21] |
|  | *C. fragile* | [19] | - | - |
|  | *E. muricatum* | [19] | - | - |
|  | *L. distichophylla* | [19] | - | - |
|  | *U. pertusa* | [19] | - | - |
|  | *R. sphaeroides* | [19] | - | - |
|  | *S. ansochromogenes* | [19] | - | - |
|  | *S. griseovariabilis* | [19] | - | - |
|  | *E. scoparia* | [19] | - | - |
|  | *H. erinaceus* | [19] | - | - |
|  | *P. citreonigrum* | [19] | - | - |
|  | *P. lagenophorae* | [19] | - | - |
|  | *S. araneosa* | [19] | - | - |
| Cymenes | [*S. griseovariabilis*](https://www.amibase.org/detail_data.php?scientific_name=Streptomyces_griseovariabilis) | [15]  Found in soil and water in Thailand [19] | - | - |
|  | *L. distichophylla* | [19] | - | - |
|  | *U. clathrate* | [19] | - | - |
|  | *U. pertusa* | [19] | - | - |
|  | *B. hyodysenteriae* | [19] | - | - |
|  | *B. laterosporus* | [19] | - | - |
|  | *C. biazotea* | [19] | - | - |
|  | *R. phaseoli* | [19] | - | - |
|  | *R. sphaeroides* | [19] | - | - |
|  | *R. vannielii* | [19] | - | - |
|  | *S. althioticus* | [19] | - | - |
|  | *S. lavendulae* | [19] | - | - |
|  | *S. tumescens* | [19] | - | - |
|  | *S. violaceochromogenes* | [19] | - | - |
|  | *A. hylecoeti* | [19] | - | - |
|  | *A. triseptatus* | [19] | - | - |
|  | *A. tabescens* | [19] | - | - |
|  | *A. ruber* | [19] | - | - |
|  | *B. murorum* | [19] | - | - |
|  | *C. aculeata* | [19] | - | - |
|  | *C. evansii* | [19] | - | - |
|  | *C. tenuissimum* | [19] | - | - |
|  | *c. rosea* | [19] | - | - |
|  | *E. scoparia* | [19] | - | - |
|  | *L. scrobiculatus* | [19] | - | - |
|  | *L. giganteus* | [19] | - | - |
|  | *P. citreonigrum* | [19] | - | - |
|  | *P. guttulate* | [19] | - | - |
|  | *R. elegans* | [19] | - | - |
|  | *R. queletii* | [19] | - | - |
|  | *S. araneosa* | [19] | - | - |
|  | *S. sasakii* | [19] | - | - |
|  | *S. granulatus* | [19] | - | - |
|  | *U. pustulata* | [19] | - | - |
|  | *U. floridaProtozoa* | [19] | - | - |
|  | *C. fruticulose* | [19] | - | - |
| Benzeneacetaldehyde | *E. coli* | [23] | - | - |
|  | *H. erinaceus* | [19] | - | - |
|  | *P. impudicus* | [19] | - | - |
|  | *S. cerevisiae* | [19] | - | - |
| Acetophenone | *S. maltophilia* | [22] | - | - |
| α,α-dimethyl-benzenemethanol | *S. maltophilia* | [22] | - | - |

*Benzene derivative and the related volatile markers for COVID-19 screening*

In order to effectively discriminate the COVID-19 positive and negative sweat samples, several cutoff area thresholds of the total and individual monoaromatic compounds as well as the potential markers, *in preparation*, in GC-MS in analysis of 164 samples obtained from Chulalongkorn hospital were varied to construct the ROC curve. All the analyzed samples were within the period of alpha and beta variants. The collected COVID-19 positive or COVID-19 negative samples were validated with "RT-PCR detected" or "RT-PCR not detected", respectively. True positive (TP), false positive (FP), true negative (TN), false negative (FN), sensitivity ratio = TP/(TP+FN) and specificity ratio = TN/(TN+FP) ratio were calculated for all the threshold values in order to obtain an optimal one. To this end, each ROC curve was generated by plotting between TP rate (or sensitivity, y-axis) and FP rate (or 1-specificity, x-axis) which can represent the COVID-19 screening performance at different threshold. The cutoff ratio was adjusted according to the research goal. For example, the cutoff value for the portable device in this study can be adjusted by changing the filter types and amounts. The ROC curves for the total monoaromatic compound peak areas and that of the individual compounds were illustrated in **Fig. S1**. At the optimum threshold, measurement of the total aromatic content could discriminate the Covid-19 populations as with the sensitivity of 96% and specificity of 33% (78% accuracy), **Fig. S1A**. This indicates insufficient capability (*e.g.* of direct PID analysis applied at 9.8 eV in this study without selective filtration detecting total aromatic content and the related compounds with the similar or lower ionization potentials such as ketones, esters, aldehydes or amines) for practical application requiring >90% for each parameter. However, the screening performance could be improved by considering the individual compounds. This could lead up to the 92% sensitivity, specificity and accuracy for *p*-cymene (**Fig. S1C**). Combination of more than one compounds could further improve the screening results, *e.g.* up to for the features of acetophenone//(E)-2-octenal//1-chloro-octane and 2-ethylhexyl acrylate (**Fig. S1F**). This analysis approach shows a potential for further development in a portable PID platform coupled with an effective filter showing selectivity towards the potential marker compounds.

**Fig. S1.** ROC curves including classification rate indices of sensitivity (Sen), specificity (Spe) and accuracy (Acc) of the different sets of potential marker compounds for armpit sweat samples and that obtained from the portable result. The dots show the optimal cutoff thresholds of normalized peak areas or the portable sensor response to discriminate the COVID-19 patients during the period of alpha and beta variants.

*Application of the developed sensor for screening of COVID-19 positive populations in Bangkok, Thailand*

In this section, the collected COVID-19 positive samples; RT-PCR detected or rapid antigen test positive and COVID-19 negative samples; RT-PCR not detected or asymptomatic and rapid antigen test negative, were validated. The related numbers were 64 positive & 61 negative, 156 positive & 357 negative, 684 positive & 6424 negative and 361 positive & 127 negative samples during the periods of research phase, Alpha-Beta, Delta and Omicrons variant of SARS-CoV-2 outbreak, respectively. They were investigated with the portable PID-*Filter B* with the signal distributions plotted **in Fig. 3** and the ROC curves showing the performances provided in **Fig. S2**. This indicates >97% accuracies of the developed approach for screening of the investigated COVID-19 samples in Bangkok.

**Fig. S2**. ROC and classification rate indices for the COVID-19 screening tests using the developed PID-*Filter B* during the periods of different variants. The dots show the optimal cutoff thresholds of the PID responses to discriminate the COVID-19 patients.

**Fig. S3**. Plots of responses measured by the developed PID-*Filter B* vs the number of repeated tests for the positive control 1 (•) and the positive control 2 (**×**) performed within every set of 10-20 sample tests during the CO0VID-19 screening. The repeated tests for all the negative controls showed zero responses.

False positive results could be observed due to several reasons. The already presence of cutaneous microbiomes producing aromatic compounds that are not caused by the SARS-CoV-2 infection. For example, *B. epidermidis* or *S. epidermidis* [6] could generate toluene [6] or ethylbenzene [8], respectively. *P. aeruginosa* in the wounded skins [9] could produce styrene [22]. High phenylalanine content in bodies caused by some disease, such as phenylketonuria, or presence of cinnamic acids in personal care products, *e.g.* perfume on the skin [24], could also be metabolized onto styrene [12]. In addition, local microbiome such as *S. griseovariabilis* involving *p*-cymene production could be observed in soil and water in Thailand [19]. These aromatic compounds could be detectable with significant recoveries using the PID-*Filter C* (**Fig. 2**) which led to false positive screening result in our study which could be found in the population such as a few persons with specific perfume, not cleaning the bodies for several days, or being microbiologists or workers with dirt. People with the positive results are thus needed to be interviewed prior further medical treatment in order to prevent false positive identification. Interestingly, a few persons in the close contact with the COVID-19 positive fellow also showed the positive signals. However, they had not got the positive signals within 14 days after the contact.

Observation of false negative samples could also be explained in several aspects. Incomplete sampling was our initial problem where people performed incorrect sampling, *e.g.* outside the shirt, or with cotton rod loss, too short sampling time or loosely closed sample vials. We improved this in the later stage by increasing our staffs, letting them pay more attention to the sampling process and providing the screened persons the video clip illustration. A few persons with absolute hygiene environment, just after a shower, or intense level of deodorants in the morning. This problem could be improved by performing the screening tests in the afternoon. Furthermore, several factors could affect aromatic contents in sweat. Volatile monoaromatic hydrocarbons could further undergo several microbial pathways to result in a range of oxygenated compounds, *e.g.* anaerobic BTEX metabolism into catechol derivatives, anaerobic BTEX metabolism into benzoyl-CoA by a system of peripheral pathway enzymes [25], toluene aerobically metabolized to 4-hydroxy benzoate/4-hydroxy-2-oxopentanoate, ethylbenzene to styrene/4-hydroxy-2-oxopentanoate, *o*- or *m*-xylene to 4-hydroxy-2-oxopentanoate and styrene to acrylate/homogentisate/4-hydroxy-2-oxopentanoate [26] as well as catabolic conversion of [*p*-cymene](http://eawag-bbd.ethz.ch/servlets/pageservlet?ptype=c&compID=c0375) to *p*-cumic alcohol in *Pseudomonas putida* [27]. For oxygenated benzene derivatives, benzaldehyde could be metabolized into benzoate in *Pseudomonas putida* [28]. Acetophenone could also be converted into [benzoyl acetate](http://eawag-bbd.ethz.ch/servlets/pageservlet?ptype=c&compID=c0267) or 2-hydroxyacetophenone by acetophenone carboxylase or naphthalene 1,2-dioxygenase, respectively [29]. These pathways above could lead to reduction of sensitivity and false negative signals in the screening.

**REFERENCES:**

[1] Bethesda (MD), National Institutes of Health (US), (2021-2023)1-455.

[2] M.Y. Lai, P.K.C. Cheng, W.W.L. Lim, Clin. Infect. Dis. 41(2005) e67-71.

[3] Thailand patent no. 2201004103

[4] P. W. Lawrence, P. McKnight, Microb Biotechnol. 11(2018) 429–431.

[5] J. A. Cusumano, A. C. Dupper, Y. Malik, E. M. Gavioli, J. Banga, A. B. Caban, D. Nadkarni, A. Obla, C. V. Vasa, D. Mazo, D. R. Altman, Open Forum Infect Dis. 7(2020) ofaa518.

[6] O. V. Niels, A. Rob, G. Ulrike, B. K. Gabriella, S. Stefan, T. Willem, J. A. L. Joop, S. Gosse, C. S. Renate, PLoS One. 5(2010) e15829.

[7] C. L. Marie, N. Janette, D. Mathias, P. Robert, P. Birgit, Nucleic Acids Research. 42(2014) D744-D748.

[8] L. J. Carrie, D. B. Heather, Metabolites. 10(2020) 347.

[9] B. Wu, DermNet. 2021; https://dermnetnz.org/topics/pseudomonas-skin-infections

[10] Q. Jiuxin, C. Zhao, L. Yumei, D. Xiangke, H. Shuhong, L. Jihong, Z. Yuao, J. Zhaofang, Z. Yingdan, Z. Chao, L. Yang, L. Yingxia, L. Lei, Y. Liang, Front Cell Infect Microbiol. 11(2021) 641920.

[11] D. O. Jeffrey, M. C. Tom, N. Jessica, A. L. Joshua, N. Hojung, M. F. Adam, Ø. P. Bernhard, Mol Syst Biol. 7(2011) 535.

[12] S. Traxler, A-. C. Bischoff, R. Saß, T. Phillip, G. Peter, B. Beate, S. Theresa, K. Claudia, B. Ulrike, S. Charlotte, M. Wolfram, K. S. Jochen, Sci Rep. 8(2018) 14857.

[13] E. Danilo, R. Federica, N. Antonella, F. Pasquale, V. Francesco, Appl Environ Microbiol. 75(2009) 1990–2001.

[14] D. Huccetogullari, Z. W. Luo, S. Y. Lee, Microb Cell Fact. 18(2019) 41.

[15] https://www.amibase.org/compounds.php

[16] S. Chuachaina, I. Thaveesangsakulthai, P. Sinsukudomchai, P. Somboon, J. Traipattanakul, P. Torvorapanit, K. Chatdarong, C. Kulsing, T. Nhujak, ChemistrySelect [Submitted, ChemistrySelect]

[17] https://www.amibase.org/detail_data.php?scientific_name=Streptomyces_griseovariabilis

[18] P. W. Lawrence, P. McKnight, Microb Biotechnol. 11(2018) 429–431.

[19] Z. Huanzi, W. Yanqun, S. Zhun, Z. Lu, R. Huahui, H. Weiqun, Z. Zhaoyong, Z. Airu, Z. Jingxian, X. Fei, Y. Fangming, L. Tianzhu, Y. Feng, Z. Bei, R. Shicong, G. Mian, Z. Jiahui, L. Fang, L. Fuqiang, W. Daxi, L. Jiandong, R. Peidi, Z. Shida, Y. Huanming, W. Jian, K. Karsten, M. T. Hein, C. Weijun, Z. Nanshan, X. Xun, L. Yi-min, L. Junhua, Z. Jincun, Cell Discov. 7(2021) 23.

[20] L. Louise, L. Benjamin, B. Vadsala, S. L. Wei, J Infect. 81(2020) 266-275.

[21] Z. Xiaojuan, G. Yiyue, W. Tao, Z. Kangchen, C. Yin, W. Bin, Z. Fengcai , Z. Baoli, C. Lunbiao, Virus Res. 285(2020) 198005.

[22] N. Yusuf, A. Zakaria, M. I. Omar, A. Y. M. Shakaff, M. J. Masnan, L. M. Kamarudin, N. A. Rahim, N. Z. I. Zakaria, A. A. Abdullah, A. Othman M. S. Yasin, BMC Bioinformatics. 16(2015) 158.

[23] P. George, T. Erica, W. H. C, T. Michelle, E. Jason, G. Alan, J Chromatogr B Analyt Technol Biomed Life Sci. 877(2009) 2011-2018.

[24] B. Hadorn, F. Hanimann, P. Anders, H. C. Curtius, R. Halverson, Nature. 215(1967) 416-417.

[25] Honeywell filter report: The PID Handbook: Theory and Applications of Direct-Reading Photoionization Detectors

[26] C. S. Letizia, J. Cocchiara, A. Lapczynski, J. Lalko, A. M. Api, Food Chem Toxicol. 43(2005) 925-943.

[27] http://eawag-bbd.ethz.ch/BTEX/BTEX_map.html#anaerobic

[28] http://eawag-bbd.ethz.ch/meta/meta_map.html

[29] R. W. Eaton, J Bacteriol. 178(1996) 1351-1362.
